# Cellular-Level Versus Receptor-Level Response Threshold Hierarchies in T-Cell Activation

**DOI:** 10.3389/fimmu.2013.00250

**Published:** 2013-09-05

**Authors:** Hugo A. van den Berg, Kristin Ladell, Kelly Miners, Bruno Laugel, Sian Llewellyn-Lacey, Mathew Clement, David K. Cole, Emma Gostick, Linda Wooldridge, Andrew K. Sewell, John S. Bridgeman, David A. Price

**Affiliations:** ^1^University of Warwick, Coventry, UK; ^2^Institute of Infection and Immunity, Cardiff University, Cardiff, UK; ^3^Vaccine Research Center, National Institute of Allergy and Infectious Diseases, National Institutes of Health, Bethesda, MD, USA

**Keywords:** T-cell activation, T-cell cross-reactivity, T-cell receptor

## Abstract

Peptide-MHC (pMHC) ligand engagement by T-cell receptors (TCRs) elicits a variety of cellular responses, some of which require substantially more TCR-mediated stimulation than others. This threshold hierarchy could reside at the receptor level, where different response pathways branch off at different stages of the TCR/CD3 triggering cascade, or at the cellular level, where the cumulative TCR signal registered by the T-cell is compared to different threshold values. Alternatively, dual-level thresholds could exist. In this study, we show that the cellular hypothesis provides the most parsimonious explanation consistent with data obtained from an in-depth analysis of distinct functional responses elicited in a clonal T-cell system by a spectrum of biophysically defined altered peptide ligands across a range of concentrations. Further, we derive a mathematical model that describes how ligand density, affinity, and off-rate all affect signaling in distinct ways. However, under the kinetic regime prevailing in the experiments reported here, the TCR/pMHC class I (pMHCI) dissociation rate was found to be the main governing factor. The CD8 coreceptor modulated the TCR/pMHCI interaction and altered peptide ligand potency. Collectively, these findings elucidate the relationship between TCR/pMHCI kinetics and cellular function, thereby providing an integrated mechanistic understanding of T-cell response profiles.

## Introduction

T lymphocyte antigen receptors mediate adaptive immune responses via interactions with disease-associated peptide ligands presented on the surface of target cells by major histocompatibility complex (MHC) molecules. In the case of CD8^+^ T-lymphocytes (CTLs), which constitute the classical T-cell effector subset, the clonotypically expressed T-cell receptors (TCRs) engage specific peptide-MHC class I (pMHCI) molecules to elicit several functions that are instrumental in eliminating the pathogenic threat (1).

The six hyper-variable complementarity-determining regions (CDRs) of the TCR govern molecular interactions with pMHCI (2). These CDRs confer the specificity of molecular recognition, allowing CTLs to attack diseased cells without causing undue harm to healthy cells (1). Nonetheless, a certain degree of degeneracy within the antigen recognition system is unavoidable (3). Indeed, ample experimental evidence supports the notion that a single TCR clonotype can interact productively with numerous peptide ligands, which typically vary in their ability to elicit different types of cellular response (4–14). Furthermore, some cellular responses are more readily evoked than others (15–18). It seems reasonable to explain this phenomenon by postulating a hierarchy of thresholds. Such a hierarchy may reside either at the level of individual TCR/CD3 complexes or at the cellular level. In the former scenario, the threshold parameters relate to distinct components of the TCR triggering process, with different responses being elicited at different stages in the development of a mature signalosome. Essentially, this is the kinetic discrimination model proposed by Rabinowitz et al. (19), whereby early responses require a shorter TCR/pMHCI dwell-time than late responses. This model is logically distinct from the kinetic proofreading model that accounts for the existence of dwell-time thresholds *per se* (20).

**Figure 1:**
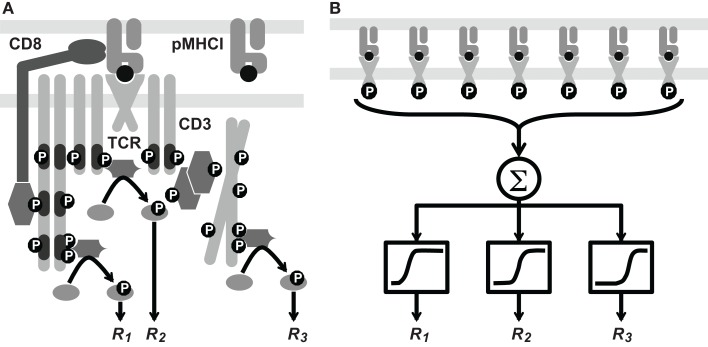
**Two hypotheses on the level of the response hierarchy**. **(A)** Signaling cascades are initiated at different points along the TCR/CD3 reaction pathway, which includes phosphorylation events and the docking of kinases and linker proteins. Thus, responses *R*_1_, *R*_2_, and *R*_3_ would correspond to distinct values of the TCR triggering threshold, i.e., $T_{R}^{\left[1\right]}<T_{R}^{\left[2\right]}<T_{R}^{\left[3\right]}$. **(B)** Cellular integration of signals from the triggered TCRs is shown as a summation (Σ) box. This signal passes through a non-linear threshold that determines whether the cell will respond. Here, *R*_1_, *R*_2_, and *R*_3_ represent various cellular responses, such as the expression of different cytokine species. Each receives the same integrated signal *W* as an input, but different values of *W* are required to initiate a response. This is depicted schematically by sigmoid curves whose midpoint lies to the left, in the middle, or to the right. These differences correspond to distinct values of the cellular activation threshold, i.e., $W_{\mathrm{act}}^{\;\left[1\right]}<W_{\mathrm{act}}^{\;\left[2\right]}<W_{\mathrm{act}}^{\;\left[3\right]}$. These two hypotheses are not mutually exclusive.

In the cellular-level scenario, TCR triggering delivers a stereotypical signal that elicits distinct responses across a single quantitative gradient (e.g., concentration of the relevant signaling factor). The contrast between these two scenarios is illustrated diagrammatically in Figure 1. A third possibility is that the hierarchy comprises a combination of both receptor-level and cellular-level modes. It has hitherto been unclear which of these three alternatives prevails in T-cell signaling.

In this study, we used a mathematical model to investigate the extent to which each of the three possibilities (receptor-level hierarchy, cellular-level hierarchy, or a combination of both) agree with experimental data. Our model accounts for the functional sensitivity of TCR-mediated responses to a given pMHCI ligand on the basis of TCR/pMHCI interaction kinetics. The concept that TCR/pMHCI kinetics governs functional sensitivity was pioneered by Lanzavecchia et al. (21) and subsequently formulated as a mathematical model (22, 23). Bridgeman et al. (24) recently summarized the available evidence across reported systems.

Analysis of TCR/pMHCI interaction kinetics demonstrates that functional sensitivity is not dependent on a single biophysical parameter, but rather on the interplay between association rate, dissociation rate, and ligand densities (23, 25, 26). Under the experimental conditions prevailing in the present study, however, the dissociation rate (the reciprocal of the mean dwell-time of the TCR/pMHCI interaction) emerged as the dominant biophysical parameter. Moreover, we estimated the extent to which the CD8 coreceptor modulates this parameter using data from parallel experiments conducted in the absence of an extracellular MHCI/CD8 interaction.

Five distinct cellular responses were investigated: (i) mobilization of the degranulation marker CD107a; (ii) secretion of macrophage inflammatory protein 1-β (MIP-1β); (iii) secretion of tumor necrosis factor-α (TNF-α); (iv) secretion of interleukin-2 (IL-2); and (v) secretion of interferon-γ (IFN-γ). Measured simultaneously and independently by flow cytometry, the magnitude of each response was quantified as a function of fluorescence and plotted against peptide concentration. The resulting dose-response curves indicated that the five cellular responses were elicited by pMHCI stimulation according to a pronounced hierarchy. Analysis of these curves by means of the mathematical model, in conjunction with biophysical data, indicated that the cellular-level response threshold hierarchy hypothesis provides the most parsimonious explanation.

## Materials and Methods

### Experimental procedures

#### Cells and peptide ligands

The CTL clone ILA1 recognizes residues 540–548 (ILAKFLHWL in single-letter amino acid code, abbreviated as ILA) of the human telomerase reverse transcriptase protein presented in the context of the human MHCI allotype HLA-A^∗^0201 (HLA-A2). Cell culture was performed as described previously (27). The altered peptide ligands used in this study are referred to here as 3G, 5Y, 8T, 8E, and 3G8T; these ligands were largely characterized previously (27, 28), and display equivalent binding to HLA-A2 (29).

#### Bioassay

Stable C1R transfectants expressing wild-type HLA-A2 (C1R-A2) or CD8-null HLA-A2 (C1R-A2null), the α3 domain of which contains the double mutation D227K/T228A that abrogates CD8 binding (30), were pulsed with peptide as indicated for 1 h at 37°C. For each condition, assays were set up simultaneously in 96-well plates using 4.5 × 10^5^ C1R cells per well, thereby ensuring that all cellular parameters were consistent across ligand stimulations. Subsequently, brefeldin A (10 μg/ml; BD Biosciences) and monensin (0.7 μl/ml; BD Biosciences) were added together with a pre-titred concentration of the directly conjugated monoclonal antibody (mAb) αCD107a-FITC [BD Pharmingen; Ref. (31)]. Serum-starved CTLs, incubated in Roswell Park Memorial Institute (RPMI) medium (Life Technologies) containing 2% fetal calf serum (FCS) for 16 h prior to assay, were subsequently added at 9 × 10^5^ cells per well. After incubation for 6 h, which generally allows sufficient time for even the most sluggish response to appear, cells were washed in phosphate-buffered saline (PBS) containing 1% FCS and incubated for 10 min at room temperature with Aqua fixable live/dead cell stain (Life Technologies) to enable the exclusion of dead cells from the analysis. Pre-titred concentrations of αCD3-H7allophycocyanin (BD Biosciences), αCD8-QD705 (Life Technologies), and αCD19-V500 (BD Horizon) were then added for 20 min at 4°C. After two further washes in PBS/1% FCS, cells were fixed/permeabilized using a Cytofix/Cytoperm™ kit (BD Biosciences) according to the manufacturer’s instructions, then stained intracellularly with pre-titred concentrations of αMIP-1β-PE (BD Pharmingen), αIFNγ-V450 (BD Horizon), αIL-2-allophycocyanin (BD Pharmingen), and αTNFα-PECy7 (BD Pharmingen) for 20 min at 4°C.

#### Flow cytometry

Stained cell samples were acquired and recorded using a customized FACS Aria II flow cytometer (BD Biosciences) equipped for the simultaneous detection of 18 fluorescent parameters. Polychromatic analyses were conducted using FlowJo software version 9.5.2 (TreeStar Inc.). The following gating tree was applied: (i) single cells were identified based on their light scatter properties; (ii) Boolean gating was carried out to exclude artifacts and fluorochrome aggregates; (iii) viable CD3^+^CD19^−^ events were selected; (iv) outliers were eliminated in a side-scatter versus CD3 display; and (v) gates were set on cells positive for individual functional read-outs. The frequencies and median fluorescence intensity (MFI) values of each functional response were exported for data analysis to *Excel* and onward for simultaneous curve fitting in *Mathematica*.

#### Surface plasmon resonance

Soluble TCR, derived from the ILA1 CTL clone, was manufactured as described previously (32, 33). Binding analysis by surface plasmon resonance (SPR) was performed using a BIAcore T100™ equipped with a CM5 sensor chip (34). Biotinylated pMHCI (200–400 response units) was immobilized to streptavidin, which was chemically linked to the chip surface. The pMHCI was injected at a slow flow rate (10 μl/min) to ensure uniform distribution on the chip surface. Combined with the small amount of pMHCI bound to the chip surface, this reduced the likelihood of dissociation rate limiting mass transfer effects. The ILA1 TCR was purified and concentrated to ∼100 μM on the day of SPR analysis to reduce the likelihood of TCR aggregation affecting the results. At least 5 serial dilutions were prepared in duplicate and injected over the relevant sensor chips at a flow rate of 45 μl/min. All experiments were conducted at 25°C. Results were analyzed using *BIAevaluation*, *Excel*, and *Origin*.

### Theory and data analysis

#### Assumptions

The assumptions of the TCR triggering model are as follows: (i) TCRs on the T-cell surface become “triggered” (i.e., are induced to become signalosomes) during an interaction with a pMHCI ligand; and (ii) the T-cell accumulates the signals emanating from triggered TCRs over space (cell:cell interface) and time (duration of cell:cell interaction), proceeding with a response when this signal exceeds a cellular activation threshold. The mathematical formulation of these assumptions is based on the kinetics of the TCR/pMHCI interaction.

#### The TCR triggering rate equation

The model describes the kinetics of interactions between TCR and pMHCI molecules in the interface area between a T-cell and an antigen-presenting cell (APC). This area is occupied by TCRs on the T-cell side of the interaction and by pMHCI complexes on the APC side. Let *R_T_* be the total number of TCRs and *Z_T_* be the total number of pMHCI molecules. Both are subject to a conservation law:

(1) $$\begin{array}{l} R_{T}=C+R \end{array}$$

(2) $$\begin{array}{l} Z_{T}=C+Z \end{array}$$

where *C* is the number of TCR/pMHCI complexes, *R* is the number of TCRs not engaged in a complex (i.e., “free” TCR molecules) and *Z* is the number of free pMHCI molecules. Kinetic equilibrium is expressed by the law of mass action equation:

(3) $$k_{\mathrm{on}}\mathrm{RZ}=k_{\mathrm{off}}C.$$

This equation, together with equations (1) and (2), leads to a quadratic in *C* with solution

(4) $$C=\frac{R_{T}+Z_{T}+k_{\mathrm{off}}∕k_{\mathrm{on}}}{2}\left(1-\sqrt{1-\frac{4R_{T}Z_{T}}{R_{T}+Z_{T}+k_{\mathrm{off}}∕k_{\mathrm{on}}}}\right)$$

(the other root is irrelevant since it exceeds both *R_T_* and *Z_T_*). The rate at which TCRs are being triggered can be expressed as the rate at which TCR/pMHCI complexes dissociate times the probability that any given TCR/pMHCI association event results in a triggering of the associated TCR/CD3 complex:

(5) $$W=k_{\mathrm{off}}\;C\;P_{\mathrm{trig}}$$

where *W* denotes the TCR triggering rate and *P*_trig_ the triggering probability for an individual interaction event. Since triggering of a TCR/CD3 complex requires completion of a series of phosphorylation and docking events, it is reasonable to assume that triggering can only happen if the peptide ligand remains engaged for at least a certain amount of time. This minimum duration is the *TCR triggering threshold* *T_R_*. If the lifetime of the TCR/pMHCI complex is exponentially distributed, the triggering probability is given by:

(6) $$P_{\mathrm{trig}}=\;\exp\left\{-k_{\mathrm{off}}T_{R}\right\}.$$

Combined, equations (4–6) yield the general triggering rate equation:

(7) $$\begin{array}{llll} W & =\frac{k_{\mathrm{off}}\;\exp\left\{-k_{\mathrm{off}}T_{R}\right\}}{2}\left(R_{T}+Z_{T}+k_{\mathrm{off}}∕k_{\mathrm{on}}\right)\; & & \;\\ & \;\times \left(1-\sqrt{1-\frac{4R_{T}Z_{T}}{R_{T}+Z_{T}+k_{\mathrm{off}}∕k_{\mathrm{on}}}}\right).\; \end{array}$$

Simple forms in special cases [equations (13–15)] are obtained by using the first-order approximation to the square root in equation (7).

#### Connecting the theoretical model to experimental observations

The available data comprise cellular response measurements (read-outs) of T-cell activation following exposure to APCs incubated with various peptide ligands across a range of concentrations, as well as TCR/pMHCI association and dissociation rates for each peptide ligand, measured via SPR. To relate these measurements to the TCR triggering theory, a number of auxiliary assumptions are required. In particular, if *Y* is the peptide incubation concentration, the following proportionality is assumed:

(8) $$Z_{T}=\alpha Y.$$

If *K_D_* is the dissociation constant determined via SPR, the following proportionality is assumed:

(9) $$\frac{k_{\mathrm{off}}}{k_{\mathrm{on}}}=\kappa K_{D}.$$

The proportionality constant κ is required to convert rates as measured via SPR to the 2-dimensional environment of the cell:cell interaction area. To connect the model to data, two compound parameters are introduced: ζ = α/κ (dimensionless), and ρ = *R_T_*/κ (M). With these parameters, the TCR triggering rate *w* = *W*/κ (in M⋅s^−1^) assumes a simpler form:

(10) $$\begin{array}{llll} w & =\frac{k_{\mathrm{off}}\;\exp\left\{-k_{\mathrm{off}}T_{R}\right\}}{2}\left(\rho +\zeta Y+K_{D}\right)\; & & \;\\ & \;\times \left(1-\sqrt{1-\frac{4\rho \zeta Y}{\rho +\zeta Y+K_{D}}}\right).\; \end{array}$$

The read-out in the present study is median fluorescence intensity (MFI), which is a valid measure of activation in view of its good correlation with the fraction of responding cells. Accordingly, the read-out *U* is assumed to be proportional to the fraction of responding cells. This assumption is validated by response data at the level of individual cells (see Results below). The equation for the read-out is as follows:

(11) $$U=U_{\min}+\left(U_{\max}-U_{\min}\right)P\left(\mathrm{respond}\right)$$

where *U*_min_ and *U*_max_ are nuisance parameters associated with the read-out procedure; these parameters take specific values for each type of cellular response and are assumed not to vary across peptide ligands. The probability that a T-cell responds is modeled as follows:

(12) $$P\left(\mathrm{respond}\right)=P\left(w_{\mathrm{act}}\leq w\right)$$

where *w* is the scaled TCR triggering rate given by equation (10) and *w*_act_ is the *cellular activation threshold*. This latter quantity is assumed to have a log-normal distribution over the population of T-cells in the experiment. Stochastic variation between the T-cells within the responding population is thus taken into account. The parameters are estimated by simultaneous least-squares fitting over the set of available ligands and read-outs. The median of the log-normal distribution provides the estimate of *w*_act_.

#### Implementation of response hierarchy hypotheses

We examined five different cellular responses and compared three hypotheses: (i) for each type of cellular response, both *T_R_* and *w*_act_ have distinct values; (ii) there is a common value of *T_R_* across responses, whereas *w*_act_ has a different value for each response; and (iii) there is a common value of *w*_act_ across responses, whereas *T_R_* has a different value for each response. As the least-squares fit is carried out simultaneously, these hypotheses can be implemented by specifying either common or particular values for these parameters.

## Results

### Kinetic parameters and ligand numbers interact to determine the rate of TCR triggering

The triggering equation (7) reduces to various simplified forms, depending on which receptors are kinetically limiting. These forms are of immunological interest since they illuminate the controversy over which kinetic parameter primarily governs T-cell activation. First, if the term *k*_off_/*k*_on_ is much larger than both *R_T_* and *Z_T_*, which applies when both cells have introduced comparatively low numbers of ligands into the interaction area, the following approximation is accurate:

(13) $$W=R_{T}Z_{T}k_{\mathrm{on}}\;\exp\left\{-k_{\mathrm{off}}T_{R}\right\}\;\left(k_{\mathrm{off}}∕k_{\mathrm{on}}\gg \max\left\{R_{T},Z_{T}\right\}\right)\;,$$

that is, the triggering rate will be proportional to both TCR and pMHCI numbers: signaling is *affinity-limited*.

On the other hand, if the cells express numbers of ligands in the interaction area that are much larger than the 2-dimensional dissociation constant *k*_off_/*k*_on_, there are two further good approximations:

(14) $$\begin{array}{ll} W & =R_{T}k_{\mathrm{off}}\;\exp\left\{-k_{\mathrm{off}}T_{R}\right\}\;\left(k_{\mathrm{off}}∕k_{\mathrm{on}}\ll Z_{T}\right)\; \end{array}$$

(15) $$\begin{array}{ll} W & =Z_{T}k_{\mathrm{off}}\;\exp\left\{-k_{\mathrm{off}}T_{R}\right\}\;\left(k_{\mathrm{off}}∕k_{\mathrm{on}}\ll R_{T}\right);\; \end{array}$$

in the first case, signaling is *TCR-limited* and in the second, it is *MHC-limited*. These equations imply that under the receptor-limited regimes, the TCR triggering rate displays an optimum relative to *k*_off_ at the point $k_{\mathrm{off}}=T_{R}^{-1}$, whereas under the affinity-limited regime, *W* increases monotonically in *k*_on_ and decreases monotonically in *k*_off_.

**Figure 2:**
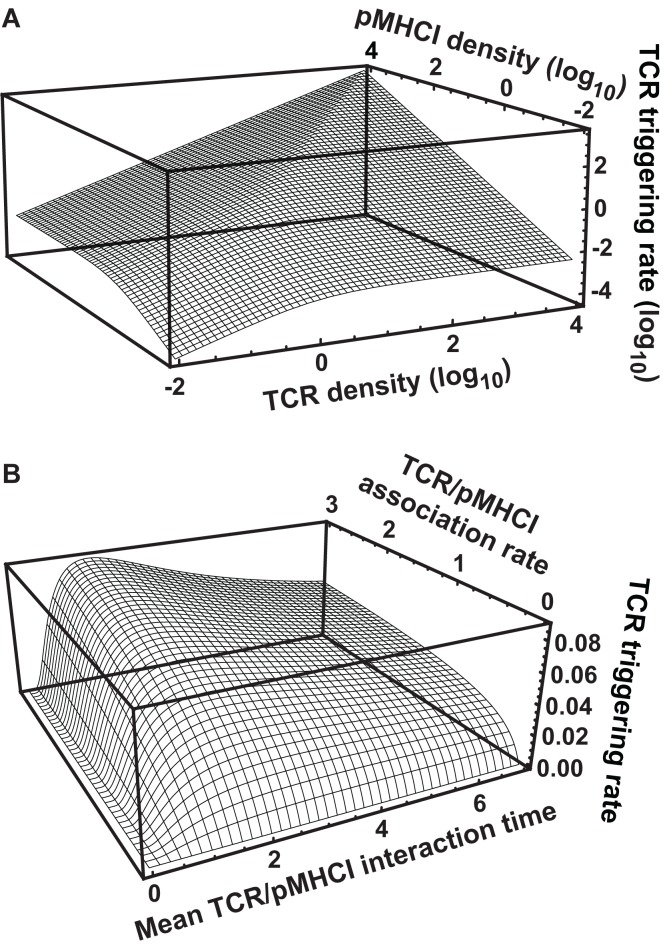
**TCR triggering rate depends on TCR/pMHCI association and dissociation rates as well as the densities of TCR and pMHCI molecules**. **(A)** Dependence of the rate of TCR triggering on receptor densities. Quantities are dimensionless: TCR triggering rate is scaled as WTR2k on, TCR density is scaled as *R_T_k*_on_*/k*_off_, pMHCI density is scaled as *Z_T_k*_on_*/k*_off_, and *k*_off_ = 1/*T_R_* is assumed (this means that the ligand is optimal under MHC-limited conditions). **(B)** Dependence of the rate of TCR triggering on kinetic parameters. The dependence on the mean TCR/pMHCI interaction time (1/*k*_off_) is non-monotone, indicating that a maximally strong agonist must satisfy *k*_off_ = 1/*T_R_*. In contrast, the dependence on *k*_on_ is monotone. Affinity is customarily expressed by the dissociation constant *K_D_ *= *k*_off_/*k*_on_. Accordingly, whether or not an improvement of affinity correlates with an enhanced TCR triggering rate depends on where the system is initially located on the surface of the graph, as well as on the relative contributions that changes in association and dissociation rates make to the overall change in affinity. Quantities are dimensionless: TCR triggering rate is scaled as *WT_R_*/(*R_T_ *+ *Z_T_*), association rate is scaled as *k*_on_*T_R_*(*R_T_ *+ *Z_T_*), and the mean lifetime of the interaction is scaled as 1/(*k*_off_*T_R_*). Here, *R_T_* = *Z_T_*. Similar qualitative behavior results when the two ligand densities are unequal.

When both *R_T_* and *Z_T_* are much larger than *k*_off_/*k*_on_, the TCR triggering rate is proportional to min{*R_T_*, *Z_T_*} as shown in Figure 2A, whereas at lower receptor densities, *W* ∝ *R_T_Z_T_* as per equation (13). The transition between the two regimes happens where the receptor densities traverse the 2-dimensional dissociation constant *k*_off_/*k*_on_. Thus, depending on receptor densities, the triggering rate may be affected solely by changes in *R_T_* (TCR copy numbers), *Z_T_* (pMHCI copy numbers), or both. The MHC-limited case corresponds to the serial triggering (serial engagement) hypothesis proposed by Valitutti et al. (35–37); however, the present theory is more general in the sense that the serial triggering mechanism arises as a special case.

Figure 2B shows that the TCR triggering rate increases with both *k*_on_ and mean dwell-time $k_{\mathrm{off}}^{-1}$ when *k*_on_ is low (relative to receptor densities; scaling is explained in the legend to Figure 2). However, when *k*_on_ increases, the triggering rate exhibits a weak dependence on *k*_on_ and a non-monotone dependence on *k*_off_, attaining a maximum at *k*_off_ = 1/*T_R_*.

The *avidity effect* is routinely exploited to determine the functional sensitivity of the TCR to a given ligand. This effect hinges on mutual compensation by pMHC ligand numbers and the TCR triggering rate per ligand; i.e., a T-cell can be activated by a strong agonist even at low copy numbers whereas a poor agonist may be effective but only at sufficiently high copy numbers. In equation (15), copy numbers are expressed by *Z_T_* and the intrinsic quality of the ligand is expressed by the quantity *k*_off_exp{−*k*_off_*T_R_*}. The latter attains a maximum at *k*_off_ = 1/*T_R_*. If the triggering rate *W* needs to exceed a certain threshold value to activate the T-cell, sufficient pMHCI copy numbers can compensate for a suboptimal intrinsic triggering rate. However, increasing *Z_T_* may transfer the system into the TCR-limited regime (Figure 2B), under which conditions pMHCI density-based compensation cannot be achieved, *cf.* equation (14). Moreover, physiological bounds must exist on how many copies can be present in the interaction area and poor agonists will be unable to activate the T-cell unless unusually high numbers of the ligand are presented (e.g., the cell transcribes very large quantities of the protein).

### Intercellular variation in response hierarchy thresholds

**Figure 3:**
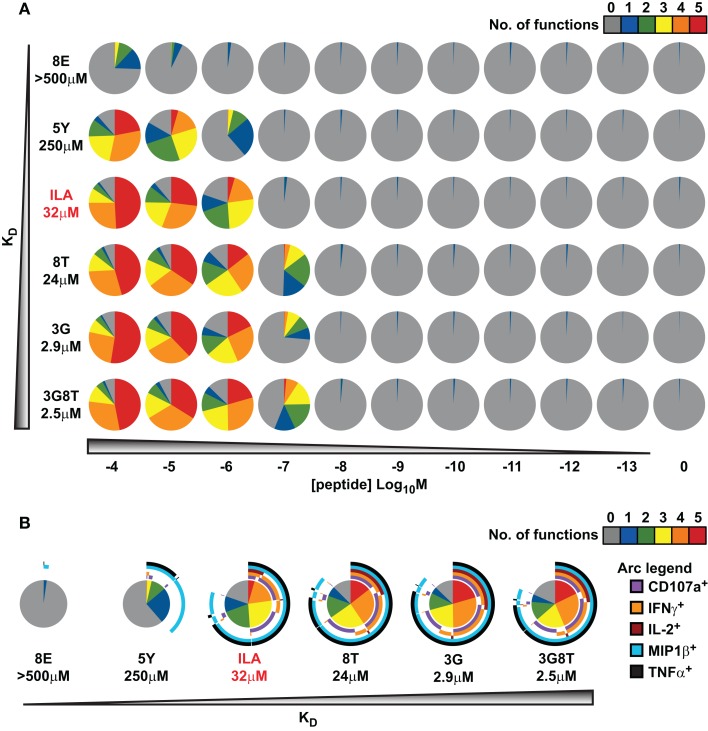
**Functional variation at the cell population level in response to six different peptide ligands**. The CTL clone ILA1 was stimulated for 6 h with peptide-pulsed C1R-A2 targets as indicated. Five functional read-outs (CD107a, MIP-1β, TNF-α, IL-2, and IFN-γ) were measured via flow cytometry. The 8E peptide was included as an extremely weak ligand. **(A)** Overview of functional profiles. Pie chart segments represent the fraction of cells expressing the number of functions indicated in the key. **(B)** Detailed analysis of functional profiles at [peptide] = 10^−6^ M. Pie charts are extended with arcs defining expressed functions as indicated in the key.

An overview of the experimental data is provided in Figure 3. In keeping with the avidity effect, the fraction of cells that exhibited at least *n* distinct responses (where 1 ≤ *n * ≤ 5) increased with peptide concentration; individual ligands differed with respect to the minimal concentration required to elicit *n* cellular responses. There was appreciable variability with regard to which functions were elicited, as depicted in Figure 3B, which displays the data obtained at peptide concentrations of 10^−6^ M. The responses generally conformed to the following series:

MIP-1β<CD107a<TNFα<IL-2<IFN-γ

(with the lowest threshold on the left). However, a small proportion of cells exhibited a slightly different ordering of threshold values, as would be expected given the natural variability between cells with respect to the threshold value of any one given response.

These results indicate that, for each response, there is variation between cells with respect to the cellular threshold value. This supports the assumption made in the section *Connecting the theoretical model to experimental observations* above, namely that the sigmoid dose-response shape of the population-level read-out can be accounted for by postulating a statistical distribution at the cell population level with respect to the cellular threshold for any given response.

### The response hierarchy resides at the level of cellular signal integration

**Figure 4:**
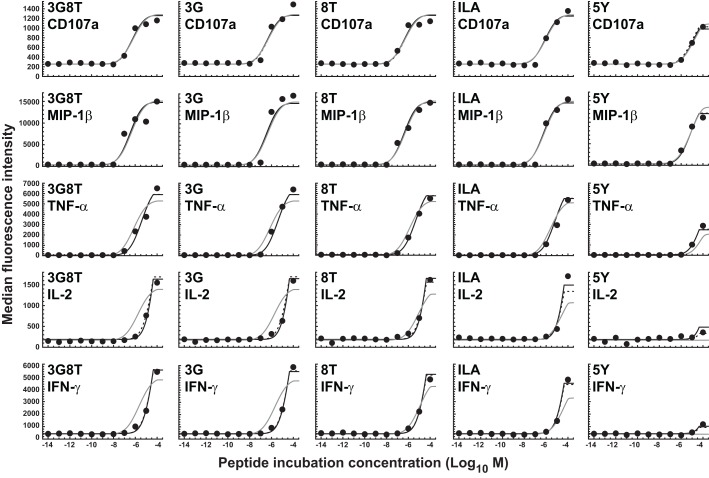
**Dose-response curves for five peptide ligands and five cellular responses**. Solid black curves correspond to the dual-threshold model in which both the receptor-level and the cellular-level thresholds have different values for different cellular responses. Solid gray curves correspond to the receptor-level threshold model in which the receptor-level thresholds have different values for different cellular responses, but the cellular activation threshold is constant. Dashed curves correspond to the cellular-level threshold model in which the cellular activation thresholds have different values for different cellular responses, but the receptor-level threshold is constant.

**Figure A1:**
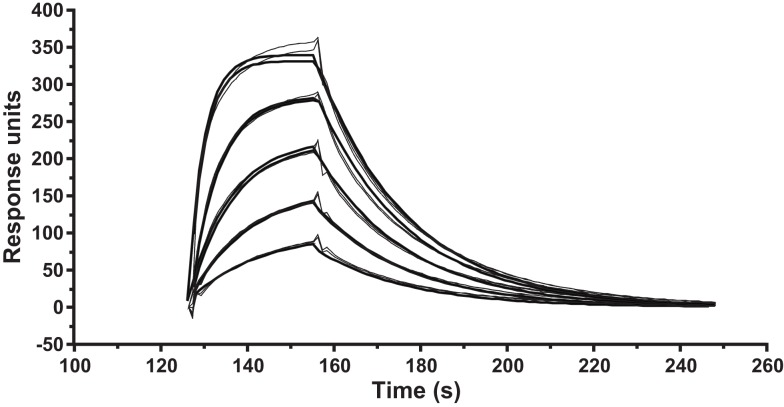
**Kinetic measurements of ILA1 TCR interactions with the agonist pMHCI ligand 3G8T**. SPR kinetic measurements were performed with five serial twofold dilutions of the ILA1 TCR, starting at 50 μM, flowed over immobilized 3G8T/HLA-A2 complexes. Injections were performed in duplicate for each TCR concentration. The plots show data (thin lines) and curve fitting (thick lines) representative of two separate experiments. First-order exponential association rate (*k*_on_) and dissociation rate (*k*_off_) were fitted simultaneously by least squares. Values for *k*_on_, *k*_off_, and *K_D_* are shown inTable 1.

The parameters of the mathematical model were estimated by means of least-squares fitting of model predictions to the data; the biophysical data are summarized in Table 1 and Figure A1 in the Appendix. Figure 4 shows the results of least-squares curve fitting for the dual-hierarchy hypothesis, the receptor-level response hierarchy hypothesis, and the cellular-level response hierarchy hypothesis. The goodness-of-fit should be assessed in the light of the parsimoniousness of the model. There is less than one parameter per curve. This compares favorably to the standard practice of fitting a separate sigmoid model to each curve, which requires three or more parameters per curve. The fit is very good for such extreme parameter-count efficiency.

**Table 1:** **Biophysical parameters**.

Ligand	On-rate (M^−1^s^−1^)	Off-rate (s^−1^)	*K_D_* (M)
3G8T	19,500	0.049	2.5 × 10^−6^
3G	16,000	0.047	2.9 × 10^−6^
8T	4,000	0.095	2.4 × 10^−5^
ILA	4,100	0.13	3.2 × 10^−5^
5Y	1,300	0.32	2.5 × 10^−4^

The kinetics of the TCR/pMHCI interaction for the 5Y and 3G8T ligands are reported here for the first time; it should be noted that the 5Y data are subject to a comparatively greater potential measurement error due to their rapidity.

There is no substantial difference in the quality of fit between the dual-threshold model and the cellular-threshold model, whereas the fit to the receptor-threshold model is consistently less good. Thus, the data are best explained by postulating a hierarchy only at the level of the cellular activation threshold.

The MIP-1β response was found to have the lowest activation threshold. Relative to this value, the ratios of median threshold values were found to be as follows: CD107a/MIP-1β = 1.44; TNFα/MIP-1β = 11.7; IL-2/MIP-1β = 225; and IFN-γ/MIP-1β = 231. These findings broadly agree with the consensus series derived from the data reported in Figure 3. The estimated median activation threshold values are markedly higher for IL-2 and IFN-γ, which required a stimulus over two orders of magnitude larger compared to the more readily elicited responses.

### MHCI/CD8 binding modulates functional sensitivity

The experiments described above were repeated using C1R-A2null cells as APCs, which cannot productively engage the CD8 coreceptor (38). It is well-attested that the MHCI/CD8 interaction modulates the TCR/pMHCI association rate (*k*_on_), the TCR/pMHCI dissociation rate (*k*_off_), and the receptor-level triggering threshold (*T_R_*) (27, 38, 39). Multipliers can be used to model these effects. For example, *k*_off_ is replaced throughout by $\gamma_{\text{off}}$*k*_off_ where $\gamma_{\text{off}}$ represents the effect of abrogating the MHCI/CD8 interaction and $\gamma_{\text{off}}$ > 1 since CD8 stabilizes the TCR/pMHCI interaction (38–40). The parameter $\gamma_{\text{off}}$ was set to 2.16, the value reported by Wooldridge et al. (38).

**Figure 5:**
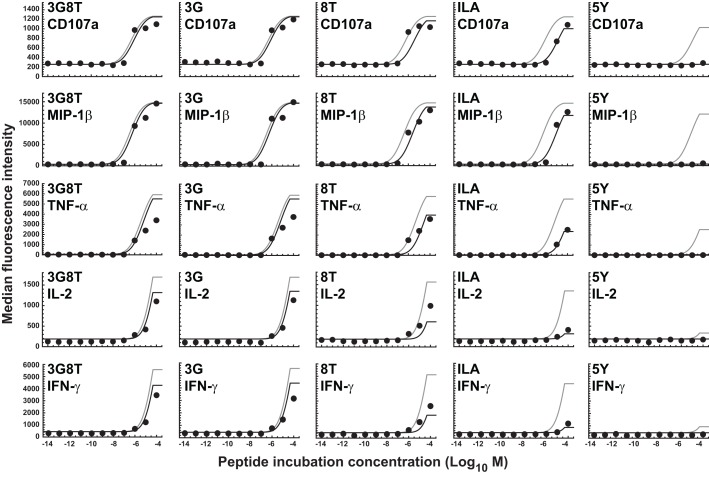
**Dose-response curves for five peptide ligands and five cellular responses in the absence of the MHCI/CD8 interaction**. All curves correspond to the cellular-level threshold model in which the cellular activation thresholds have different values for different cellular responses, but the receptor-level threshold is constant. Black curves are the fits to the data shown. Gray curves correspond to the situation in which the MHCI/CD8 interaction is present. Parameter values are as in the previous figure. Two new parameters (γ_off_ and γ_R_) have been introduced and estimated to account for the absence of the CD8 effect; γ_off_ was set to 2.16 and γ_R_ was estimated as the sole remaining free parameter.

Since the prevailing kinetic regime in the experiments described here is MHC limitation, the parameter γ_on_ for the association rate could not be estimated and was set to 1. This leaves a single free parameter, γ_R_, for the data obtained with C1R-A2null APCs. Data fits are shown in Figure 5, with γ_R_ = 1.14 (the least-squares estimate) for the receptor threshold *T_R_*. This value indicates that the MHCI/CD8 interaction decreases the TCR triggering threshold by 13%.

## Discussion

T-cells can exhibit a variety of cellular responses to TCR-mediated stimulation, some of which are elicited far more readily than others. In particular, different responses require different levels of stimulation in terms of ligand densities present on the APC surface. Such threshold differences might be postulated at the receptor level (different response pathways branch off at different stages in the sequence of signaling events), and/or at the cellular level (the TCR signal received by the T-cell needs to exceed different threshold values for different responses). The present study suggests that the cellular-level hypothesis is the most parsimonious explanation consistent with the data. Furthermore, the role of the CD8 coreceptor as a modulator of T-cell functional sensitivity is confirmed by the present findings, which indicate that the MHCI/CD8 interaction decreases the TCR/pMHCI dissociation rate as well as the duration of TCR/pMHCI contact required to activate the T-cell.

A key consideration for the interpretation of our results is the standardized nature of the system. Determinative cellular factors, such as TCR density, membrane constituency, and the expression of costimulatory/inhibitory molecules, were fixed across all conditions, which means that these extraneous factors can be discounted as discriminative because only ligand nature and concentration varied between stimulations. These built-in controls justify the simplifying assumptions from which the mathematical model was derived.

The present findings certainly do not exclude the existence of a receptor-level hierarchy. Indeed, the co-existence of hierarchies at both the receptor and cellular levels may explain the data equally well. However, the hypothesis that the response hierarchy resides solely at the receptor level is contradicted by the present findings. A threshold hierarchy at the level of cellular activation would imply that the functional responses are located downstream from signaling pathways that share a common starting point (the triggered TCR or signalosome). Further detailed molecular studies of the CD3-complex phosphorylation cascade would be required to rule out a receptor-level hierarchy definitively.

**Figure 6:**
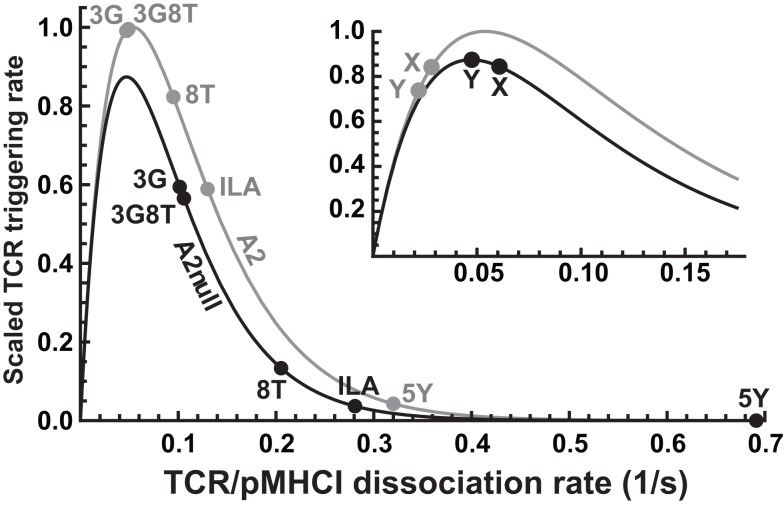
**Differential effects of CD8 modulation on functional sensitivity**. TCR triggering rate, scaled with respect to the optimal ligand under MHC-limited conditions, as a function of *k*_off_, comparing the wild-type MHCI/CD8 interaction (“A2”) to the abrogated MHCI/CD8 interaction (“A2null”). The positions for the five ligands are indicated. The inset shows two hypothetical ligands: “X,” which is unaffected by the absence of the CD8 interaction, and “Y,” which is optimal under the MHC-null condition.

**Figure 7:**
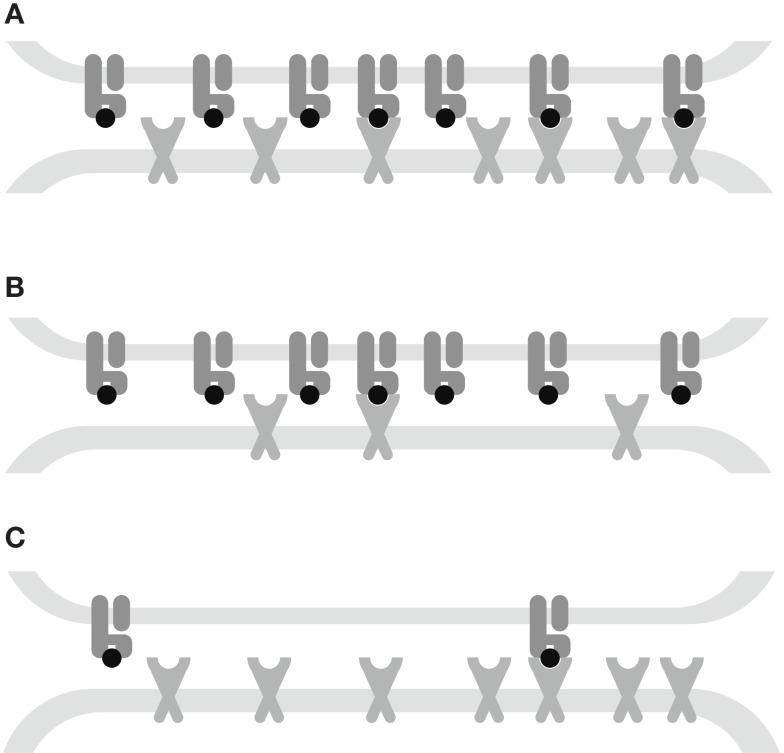
**Interplay between kinetic parameters and receptor densities**. **(A)** Affinity-limited: the TCR triggering rate is proportional to the densities of both receptors. **(B)** TCR-limited: the TCR triggering rate is proportional to the TCR density and is monotone decreasing in *k*_off_. **(C)** MHC-limited: the TCR triggering rate is proportional to the MHC density and has an optimum *k*_off_. The affinity *K_D_ *= *k*_off_/*k*_on_ governs the transitions between these kinetic regimes.

Our mathematical model elucidates the contrasting roles played by the dissociation rate and the affinity constant as determinants of T-cell functional sensitivity, with TCR and pMHCI molecular densities governing the limitation regime under which the kinetics operate. The disappearance of the optimum behavior as the system moves from the receptor-limited to the affinity-limited regime can be studied experimentally by increasing TCR density. This shift was first predicted by Van den Berg et al. (23) and confirmed experimentally by Gonzales et al. (41). Furthermore, the pattern of dependence on *k*_on_ and *k*_off_ shown in Figure 2B may account for the discrepancies between the findings of Kalergis et al. (42), who reported such an optimal dwell-time ($=k_{\mathrm{off}}^{-1}$), and the findings of Holler et al. (43), who reported monotone dependence. The shift along the *k*_on_ axis may also explain the observations of Irving et al. (26), who observed that affinity modulates the dependence on *k*_off_. In the experimental system studied here, the TCR is stimulated under MHC-limited conditions. This means that the optimum-type dependence on *k*_off_ prevails. This is shown in Figure 6, where the calculated TCR triggering rate (at 10^−5^ M) is plotted against *k*_off_. The effect of *k*_on_ is so slight that the curves overlap almost perfectly for the five ligands. Even under MHC-limited conditions, however, *k*_on_ remains a crucial determinant of functional sensitivity, in view of the fact that the ratio of *k*_off_ and *k*_on_ determines the transition between affinity-limited and ligand-limited signaling, as illustrated schematically in Figure 7. Thus, the association rate and the dissociation rate play distinct roles in T-cell activation, consistent with reports of dominant effects associated with each of these biophysical parameters under different conditions (24, 44); these differences also explain earlier observations on the interplay of kinetic parameters and receptor numbers (41, 45, 46).

In the presence of the wild-type MHCI/CD8 interaction, the ligands 3G and 3G8T are virtually optimal, whereas abrogation of MHCI/CD8 binding leads to a reduction in functional sensitivity. In virtue of the non-linear character of the curve, the magnitude of the reduction varies considerably across ligands. Two parameters (γ_on_ and γ_R_) suffice to capture these effects. The ILA ligand evokes a triggering rate approximately one order of magnitude less than optimal under C1R-A2null conditions. Nonetheless, the data show that strongly attenuated but discernible responses can be elicited at sufficiently high presentation levels, in keeping with Wooldridge et al. (14), who reported that a ligand could be physiologically significant even at an estimated ∼2.1 orders of magnitude below the optimal ligand for a responding T-cell clone.

Although all ligands studied here become less potent when presented by C1R-A2null cells, the model predicts that this is not necessarily the case. Indeed, the functional sensitivity to a ligand marked “X” in the inset of Figure 6 is unaffected when the MHCI/CD8 interaction is abrogated. Moreover, ligand “Y” becomes optimal in the absence of the CD8 effect (so that the T-cell has a higher functional sensitivity to this ligand under CD8-null conditions). The existence of such ligands would imply that T-cells can “tune” to distinct cognate ligands by up- or down-regulating their CD8 coreceptors. This effect would greatly amplify the ability of T-cells to reconcile extensive cross-reactivity with the avoidance of self-recognition.

The hypothetical ligands “X” and “Y” are strongly heteroclitic. Peptides of this nature occur markedly less frequently than homoclitic ligands, which makes them more challenging to detect and characterize. Nonetheless, work is ongoing to demonstrate the existence of such anomalous ligands, using combinatorial peptide library screening combined with importance sampling (14) in the presence of different extracellular contributions from the CD8 coreceptor (29).

The effect of CD8 on the value of the TCR triggering threshold is consistent with the conclusions of Van den Berg et al. (39) and may be due to the association of TCR/CD3 with protein tyrosine kinases such as p56lck, which expedites the immunoglobulin family tyrosine-based activation motif phosphorylation sequence (47). This agrees with the observation that the CD8 αβ hetero-dimer is more potent as a coreceptor than the αα homo-dimer (48, 49), perhaps due to interactions with other signalosome components mediated by the palmitoylated CD8 β chain (50), which interacts with myristoylated p56lck. Docking of CD8 to the TCR/CD3 complex may also be driven by initial activation (51). The kinetics of CD8 binding after TCR/pMHC contact was analyzed previously by Van den Berg and Sewell (52). More recently, Mukhopadhyay et al. (53) developed a model that explicitly accounts for the interplay between the CD3 phosphorylation cascade and p56lck, ZAP-70, and CD45.

The dissociation rates determined by SPR were assumed to be indicative of the normal interaction in this study. In reality, these values might be more true to the case where the MHCI/CD8 interaction is absent. Assuming the latter would not dramatically alter the qualitative conclusions of this study, but would lead to a slight adjustment in the interpretation of the parameter estimates. Most importantly, the value found for the TCR triggering threshold *T_R_* would be adjusted down from 17.46 to 15.26 s.

Values based on SPR experiments are best regarded as 3-dimensional, representative of ligands in solution. However, the TCR/pMHCI interaction takes place in a 2-dimensional environment, which essentially reduces spatial degrees of freedom of molecular motion and introduces dynamics related to the forces that constrain the molecules to this environment (54). Consequently, rate constants may be different in two dimensions as opposed to three dimensions. In particular, 2-dimensional dissociation rates can be substantially faster (55). The ratio between the 3-dimensional and 2-dimensional affinities is a length measure, denoted *h* and called the confinement length (56, 57). Wu et al. demonstrated that *h* is proportional to the range of motion available to the free forms of the interacting ligands along the spatial axis perpendicular to the two parallel membranes. Thus, the intermembrane separation provides an upper bound, and if the ranges of motion do not differ too much, it is reasonable to assume that the confinement length is roughly the same for all mutants involved.

The use of a fixed receptor duration might appear simplistic given the complexity of the events that are required to trigger a TCR/CD3 complex, but the law of large numbers provides indirect support for a simple assumption: the true waiting-time-till-triggering is a composite of a large number of stochastic durations which tends to regress it on the mean value. In fact, the number of terms is itself a random variate due to the possibility of alternative routes in the activation pathway of the complex (such processes are termed “compound stochastic”). The complexity illustrated in Figure 1 effectively underpins a simple assumption, viz. that there is a fixed, stereotypical receptor threshold *T_R_*.

The parameters *T_R_* and *w*_act_ are both subject to further modulation. By varying the levels of the CD8 coreceptor, surface molecules such as CD45, and cytosolic concentrations of kinases, phosphorylases, and linker proteins in the immediate vicinity of the CD3 complex, the T-cell can adjust the effective value of the receptor triggering threshold *T_R_* (29, 30, 38, 39, 58–61). The value of the cellular activation threshold *w*_act_ is modulated by costimulation (“signal 2”). Moreover, the T-cell can actively modulate its receptiveness to this signal by adjusting the relative levels of CD28 and CD152 (52, 62). Furthermore, the maintenance of tolerance requires continual dynamic tuning of the cellular activation threshold (63, 64). Conceivably, these mechanisms provide an additional “multiplier” that modulates *w*_act_ over and above the factors that set *w*_act_ to different values for different responses.

The data on intercellular response variability (Figure 3) support the assumption that the distribution of responsiveness in terms of MFI can be attributed to intercellular variation in the triggering threshold. However, the observed variability may also arise from differences in the numbers of TCR and pMHCI molecules present in the T-cell/APC interaction area. Mathematically, this model is slightly more involved because interactions can now be distributed over different kinetic regimes. Nonetheless, apart from this technical difficulty, the resulting model is mathematically equivalent; this follows from the properties of the log-normal distribution. Thus, the analysis is not substantially affected by this alternative explanation of the intercellular variability. Experimentally, the two hypotheses could be distinguished by following an individual T-cell over a series of interactions and responses. If cellular thresholds for the various responses remain largely constant, there would be little event-to-event variation according to the former hypothesis; in contrast, the receptor copy number hypothesis would predict considerable event-to-event variation.

In addition, there may be variability with respect to antigen presentation levels across the experimental APC population, due to differential antigen exposure and stochastic effects in antigen acceptance from the incubation medium. However, it can be shown that the resulting variance is not such as to make a substantial contribution to overall variability, for instance by following the argument presented in Appendix C of Van den Berg et al. (22).

When the TCR/pMHCI ternary complex dissociates, the covalent modifications and multimeric aggregations that the CD3 complex undergoes during the gradual transition to signalosome status are reversed, both by thermal agitation and the action of phosphorylases. In the present model, it has been assumed that this “reset” occurs much more rapidly than the typical time required for the TCR/CD3 complex to encounter the next non-null pMHCI ligand. However, given that the molecules have to drift away from each other through diffusion in the 2-dimensional arena of the T-cell/APC interface, there is a possibility of rapid rebinding to the *same* pMHCI molecule before the CD3 complex has had sufficient time to “reset.” This rapid rebinding is equivalent to a diminishment of the effective off-rate; a basic model capturing this effect has been proposed by Aleksic et al. (65). Extending the present model with this effect is straightforward in principle, albeit at the cost of an additional parameter. Furthermore, the number of complexes with other pMHCI species is ignored in equation (1). This is valid if the vast majority of all other ligands consists of null agonists. An analysis that takes the entire presentation profile (all pMHCI species present) into account can be found in Van den Berg et al. (22, 23).

A further aspect of the cellular activation threshold that has been left implicit in the present treatment is the duration of the T-cell/APC interaction. If this contact is initiated at *t*_0_ and is terminated at *t*_1_, the cumulative signal transmitted to the cytosolic signaling machinery is given by an integral:

(16) $$Q=\int_{t_{0}}^{t_{1}}\;W\left(\tau \right)d\tau .$$

In fact, this quantity *Q* is presumed to be directly compared to a threshold. However, Q/(*t*_1_ − *t*_0_) is proportional to the activation threshold as defined in the present study. Indeed, variability in the duration of the contact is one of the sources of stochasticity underlying the log-normal response curve used to fit the dose-response curves.

A related phenomenon is that of TCR down-regulation (66–68), whereby triggered TCRs are gradually removed from the cell surface. This affects the integral in equation (16); Van den Berg et al. (23) discuss how the model can be extended to take this effect into account. TCR down-regulation can in fact be exploited by the T-cell to gauge both *k*_on_ and *k*_off_ independently. As indicated in Figure 2A, if the system starts in the MHC-limited regime, gradual removal of TCR molecules will result in a transition to the TCR-limited regime, where a sudden drop of the instantaneous signal results. At the point of change-over, *R_T_* ≈ *Z_T_*. This means that the T-cell can, in principle, glean a rough estimate of the biophysical rate parameters *k*_off_ and *k*_on_ by combining information from the gross signal and the number of down-regulated TCRs at the transition point. This mechanism can only function in the ligand-limited regime, not in the affinity-limited regime.

In conclusion, the present study confirms that a response hierarchy exists with respect to the strength of TCR stimulation required to elicit various cellular responses. Moreover, a combination of experimentation and mathematical modeling indicates that this hierarchy resides at the cellular level rather than at the level of the individual receptor molecules. Functional sensitivity is generally enhanced by the CD8 coreceptor, although the theory indicates that the MHCI/CD8 interaction can depress TCR signaling for certain heteroclitic ligands.

## Conflict of Interest Statement

The authors declare that the research was conducted in the absence of any commercial or financial relationships that could be construed as a potential conflict of interest.
